# Crystal structure of tris­{*N*,*N*-diethyl-*N*′-[(4-nitro­phen­yl)(oxo)meth­yl]carbamimido­thio­ato}cobalt(III)

**DOI:** 10.1107/S2056989024005449

**Published:** 2024-06-11

**Authors:** Lee Roecker, Sean Parkin

**Affiliations:** a Department of Chemistry Northern Michigan University,Marquette MI 49855 USA; b Department of Chemistry University of Kentucky,Lexington KY 40506 USA; Institute of Chemistry, Chinese Academy of Sciences

**Keywords:** crystal structure, Hirshfeld surface, threefold symmetry, solvent-accessible channels

## Abstract

The synthesis, crystal structure, and a Hirshfeld surface analysis of tris­{*N*,*N*-diethyl-*N*′-[(4-nitro­phen­yl)(oxo)meth­yl]carbamimido­thio­ato}cobalt(III) conducted at 180 K are presented. The complex consists of three *N*,*N*-diethyl-*N*′-[(4-nitro­benzene)(oxo)meth­yl]carbamimido­thio­ato ligands, threefold sym­metric­ally bonded about the Co^III^ ion, in approximately octa­hedral coordination, which generates a triple of individually near planar metallacyclic (Co—S—C—N—C—O) rings. The overall geometry of the complex is determined by the mutual orientation of each metallacycle about the crystallographically imposed threefold axis [dihedral angles = 81.70 (2)°] and by the dihedral angles between the various planar groups within each asymmetric unit [metallacycle to benzene ring = 13.83 (7)°; benzene ring to nitro group = 17.494 (8)°]. The complexes stack in anti-parallel columns about the  axis of the space group (*P*), generating solvent-accessible channels along [001]. These channels contain ill-defined, multiply disordered, partial-occupancy solvent. Atom–atom contacts in the crystal packing predominantly (∼96%) involve hydrogen, the most abundant types being H⋯H (36.6%), H⋯O (31.0%), H⋯C (19.2%), H⋯N (4.8%), and H⋯S (4.4%).

## Abstract 2

The synthesis, crystal structure and a Hirshfeld surface analysis of tris­{*N*,*N*-diethyl-*N*′-[(4-nitro­phen­yl)(oxo)meth­yl]carbamimido­thio­ato}cobalt(III) are described.

## 1. Chemical context

Thio­urea derivatives and their metal complexes have been of inter­est for the past two decades. Recent reviews have highlighted current trends in their chemistry (Zahra *et al.*, 2022 ▸; Saeed *et al.*, 2014 ▸) including medical and chemosensing applications (Khan *et al.*, 2021 ▸). One older study evaluated the potential of *N*-benzoyl-*N*′-dialkyl derivatives and their Co^III^ complexes as anti­fungal agents (Wiequn *et al.*, 2003 ▸, 2005 ▸). The synthesis of these later complexes are straightforward: mixing three equivalents of the ligand with CoCl_2_·6H_2_O in water and stirring for an hour results in deposition of the neutral, dark-green Co^III^ complexes. Making the analogous tris-coordinated complexes was not our original intention. In the course of preparing Co^III^ complexes coordinated by a single κ^2−^*S*,*O* ligand, the neutral tris product was invariably formed as a side product when reacting the labile Co^III^ starting material [(en)_2_Co(OSO_2_CF_3_)]CF_3_SO_3_ (Dixon *et al.*, 1981 ▸) with one equivalent of ligand. This paper presents the synthesis and crystal structure of tris­{*N*,*N*-diethyl-*N*′-[(4-nitro­phen­yl)(oxo)meth­yl]carbamimido­thio­ato}cobalt(III), **I**.

## 2. Structural commentary

**Figure 1:**
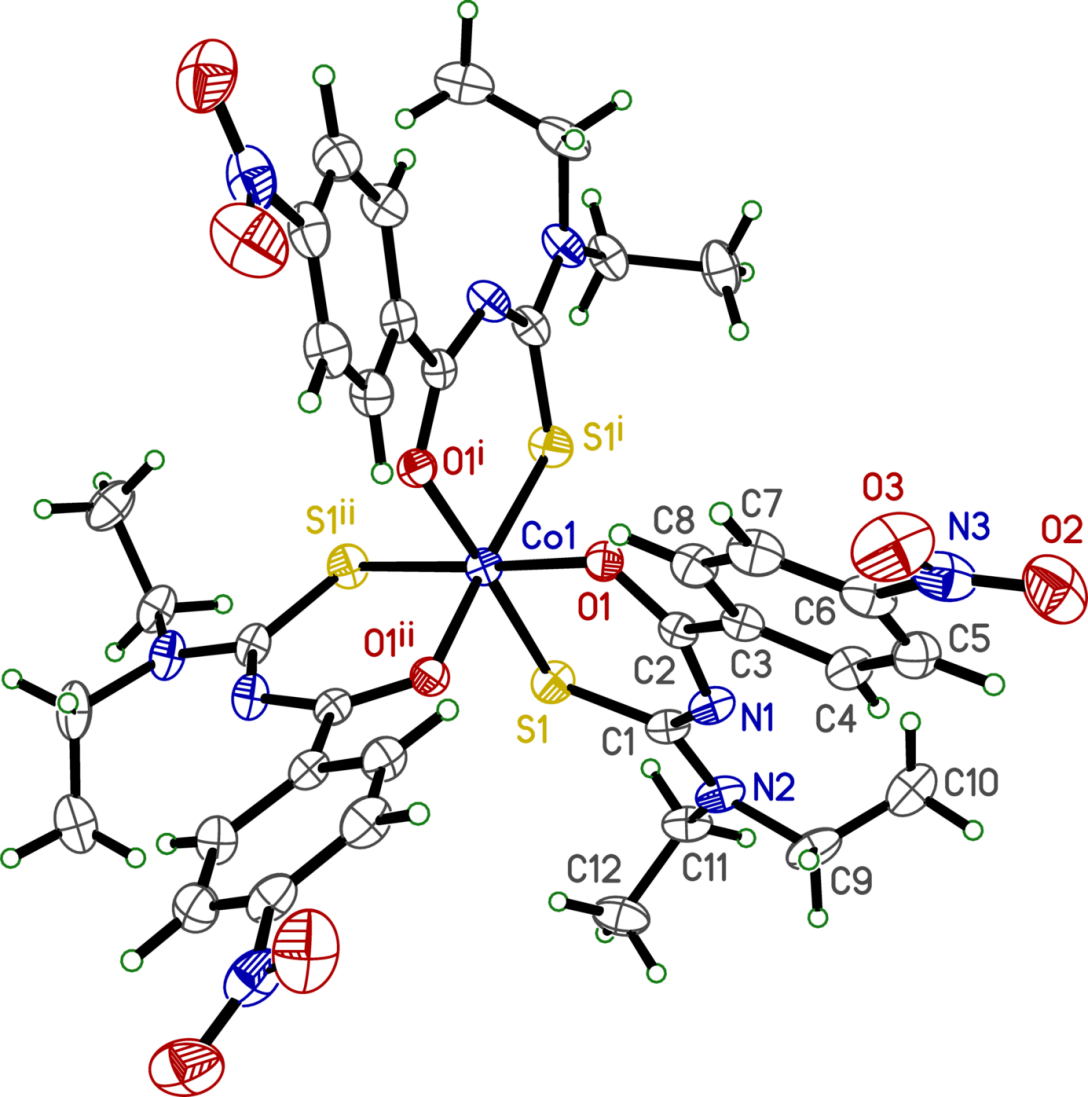
An ellipsoid plot (30% probability) of **I**. Unlabelled atoms correspond to symmetry codes: (i) −*y* + 1, *x* − *y* + 1, *z*; (ii) −*x* + *y*, −*x* + 1, *z*, as indicated by the superscripts on the O and S atoms of the symmetry-equivalent ligands.

The mol­ecule of **I** consists of three *N*,*N*-diethyl-*N*′-[(4-nitro­benzene)(oxo)meth­yl]carbamimido­thio­ato ligands, each bound to a single Co^III^ centre by their sulfur and carbonyl oxygen atoms. The complex has crystallographic threefold symmetry, with the Co^III^ atom (Fig. 1 ▸) occupying Wyckoff position *d* (1/3, 2/3, *z*) in the space group of type *P*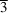. The coordination geometry about Co1 is moderately distorted octa­hedral. Due to the threefold symmetry, all Co—S bonds are equivalent [*d*_Co1—S1_ = 2.2082 (5) Å], as are all Co—O bonds [*d*_Co1—O1_ = 1.9202 (11) Å]. The bond-valence sum for Co1 amounts to 3.20 v.u. (v.u. = valence units; Brese & O’Keeffe, 1991 ▸). Deviations from octa­hedral geometry are especially apparent in the bond angles subtended at Co1. All S1—Co1—S1 [88.35 (2)°] and O1—Co1—O1 [84.68 (5)°] type bond angles are acute, while equatorial bond angles of type S1—Co—O1 [91.87 (4), 95.09 (3)] are obtuse. The axial S1—Co—O1 bond angles are 176.56 (3)°. These are summarized in Table 1 ▸.
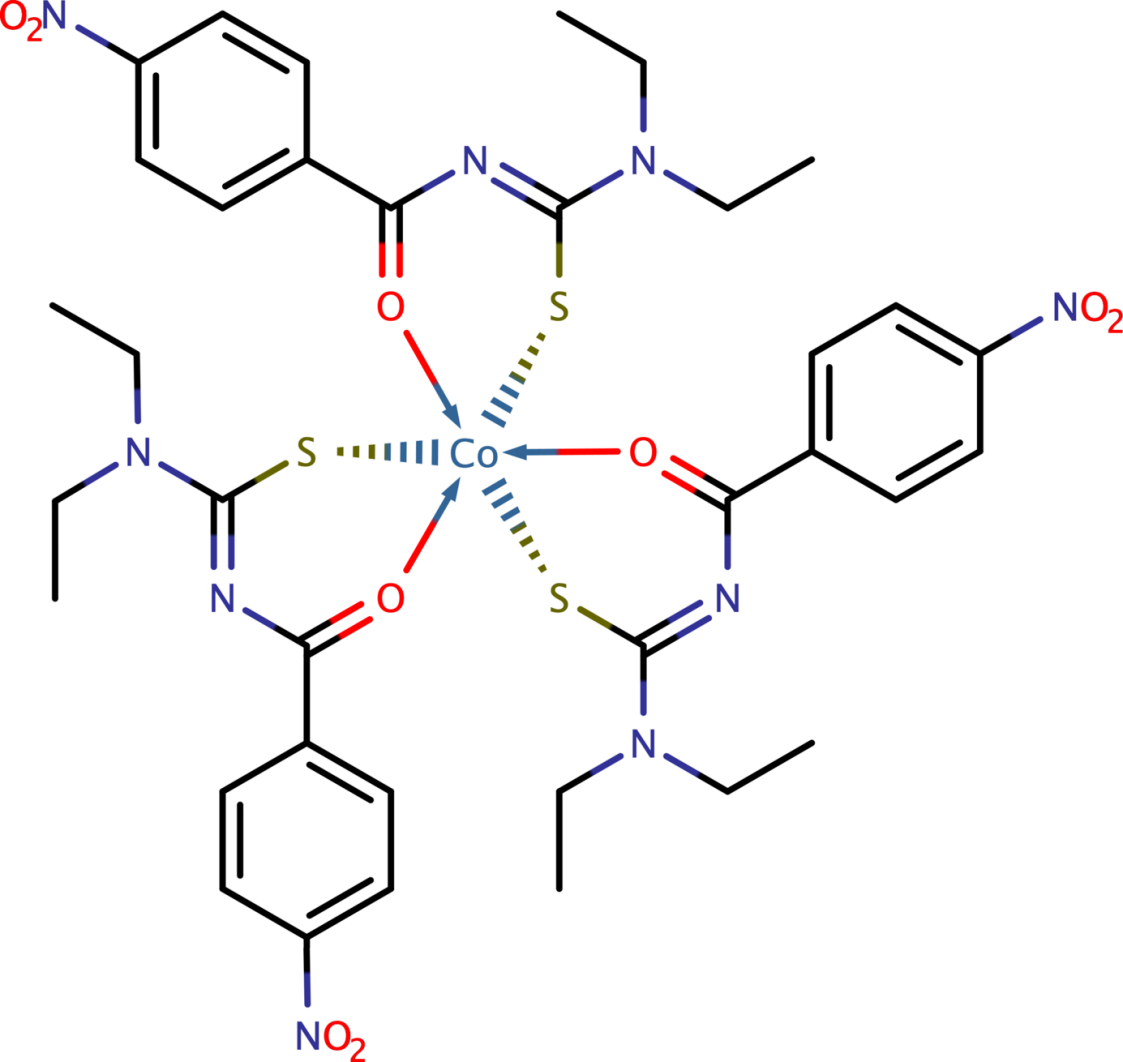


Within the asymmetric unit, the metallacyclic (Co1–S1–C1–N1–C2–O1) ring is almost planar [mean plane r.m.s. deviation = 0.0300 Å, largest = 0.0614 (12) Å at N1]. The dihedral angle between this metallacycle and the benzene ring is 13.83 (7)°, while that of the nitro group relative to the benzene ring is 17.494 (8)°. The mean plane through atoms N2–C1–C9–C11 [r.m.s. deviation = 0.0141 Å, largest = 0.0244 (14) Å at N2] forms a dihedral with the metallacycle mean plane of 6.21 (11)°. Lastly, the torsion angles of the two ethyl groups (C1—N2—C9—C10 and C1—N2—C11—C12) are the same at 90.7 (2)°. The overall geometry of the complex is then determined by the dihedral angles between the metallacycles in each asymmetric unit about the crystallographic threefold axis, which are all symmetrically equivalent at 81.70 (2)°.

## 3. Supra­molecular features

**Figure 2:**
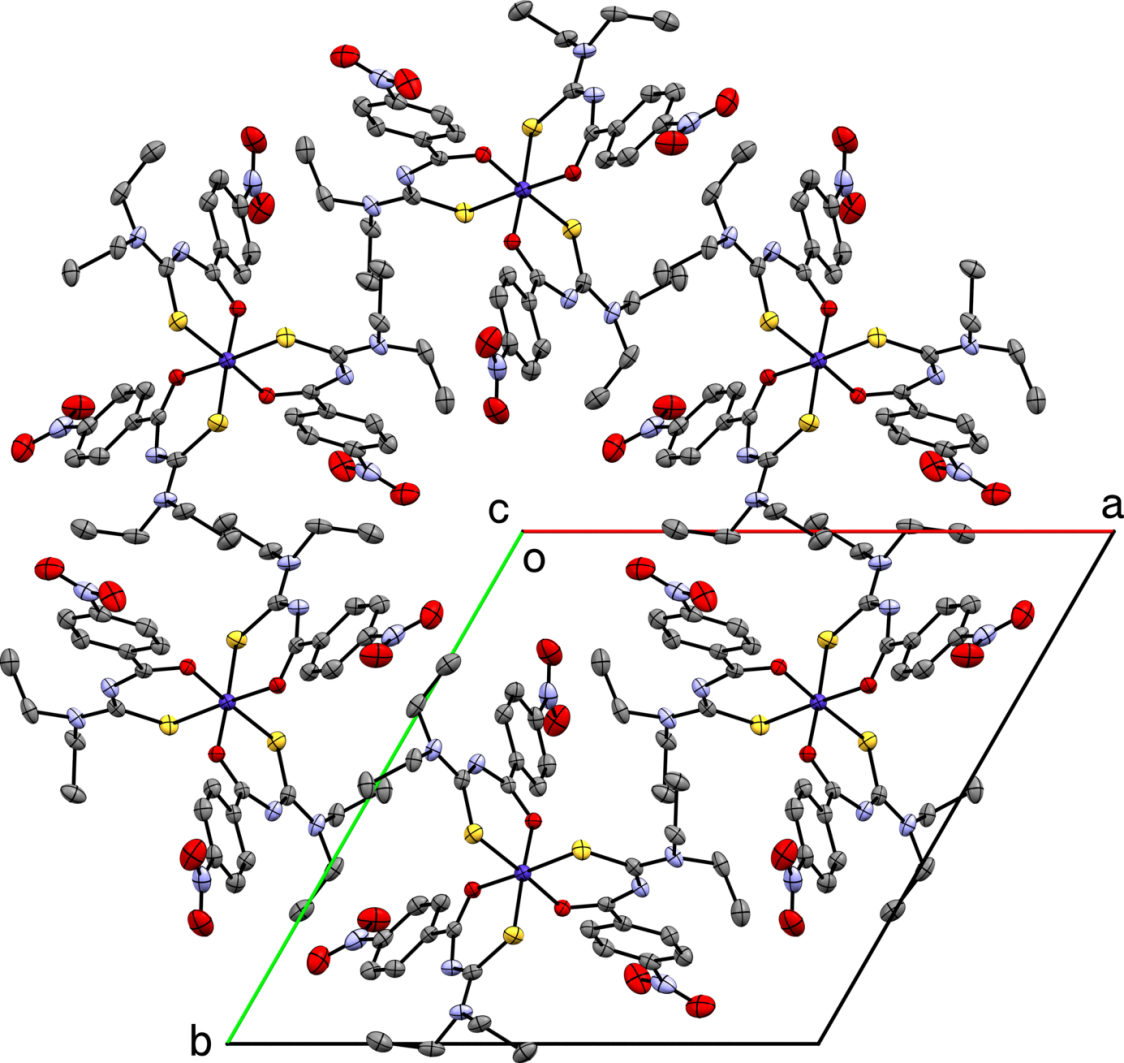
A packing plot of **I** viewed down [001], showing the extended channels running through the crystal along the *c*-axis direction.

**Figure 3:**
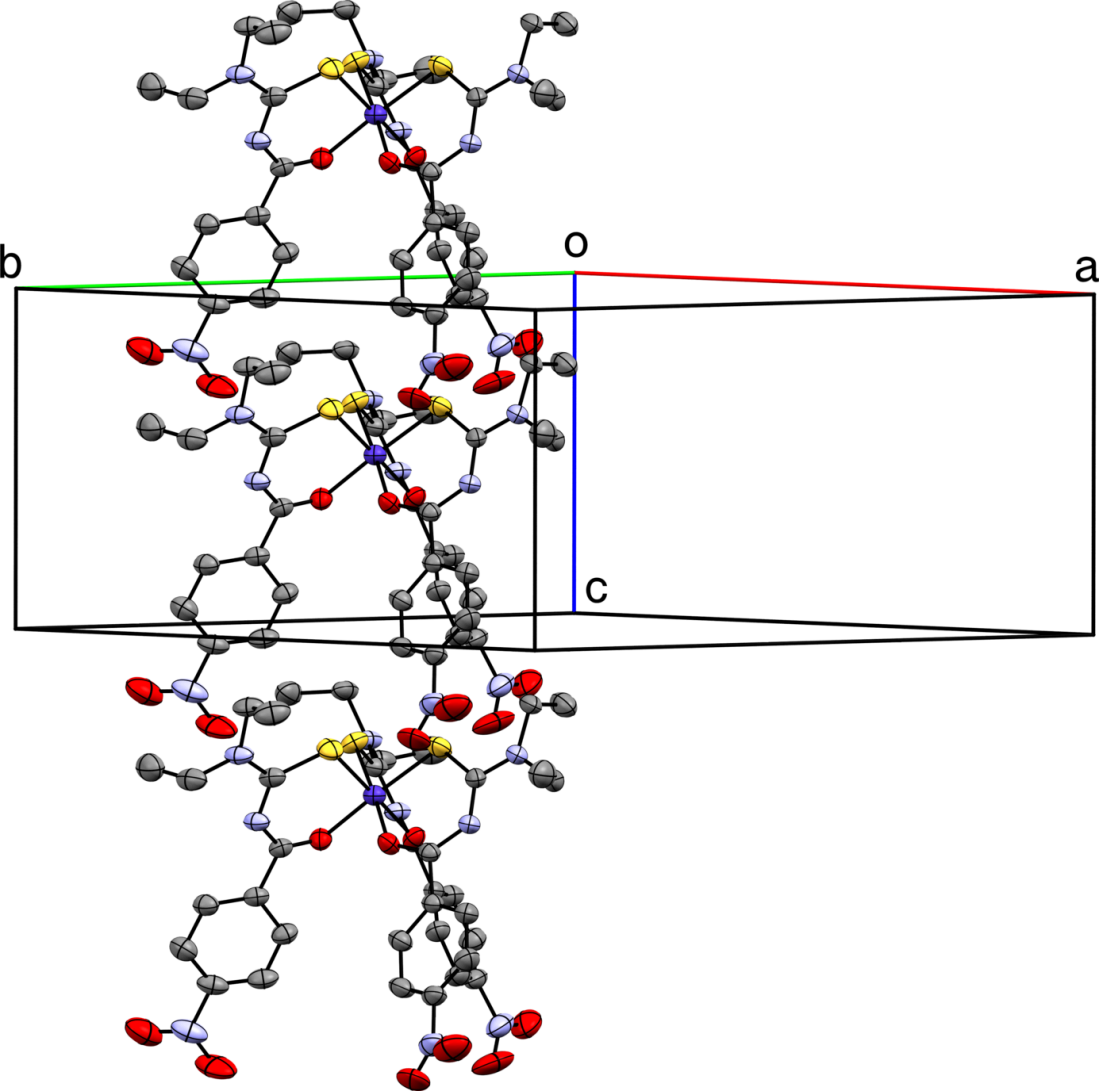
A partial packing plot of **I** viewed approximately along [110] showing a column of mol­ecules extending parallel to [001].

**Figure 4:**
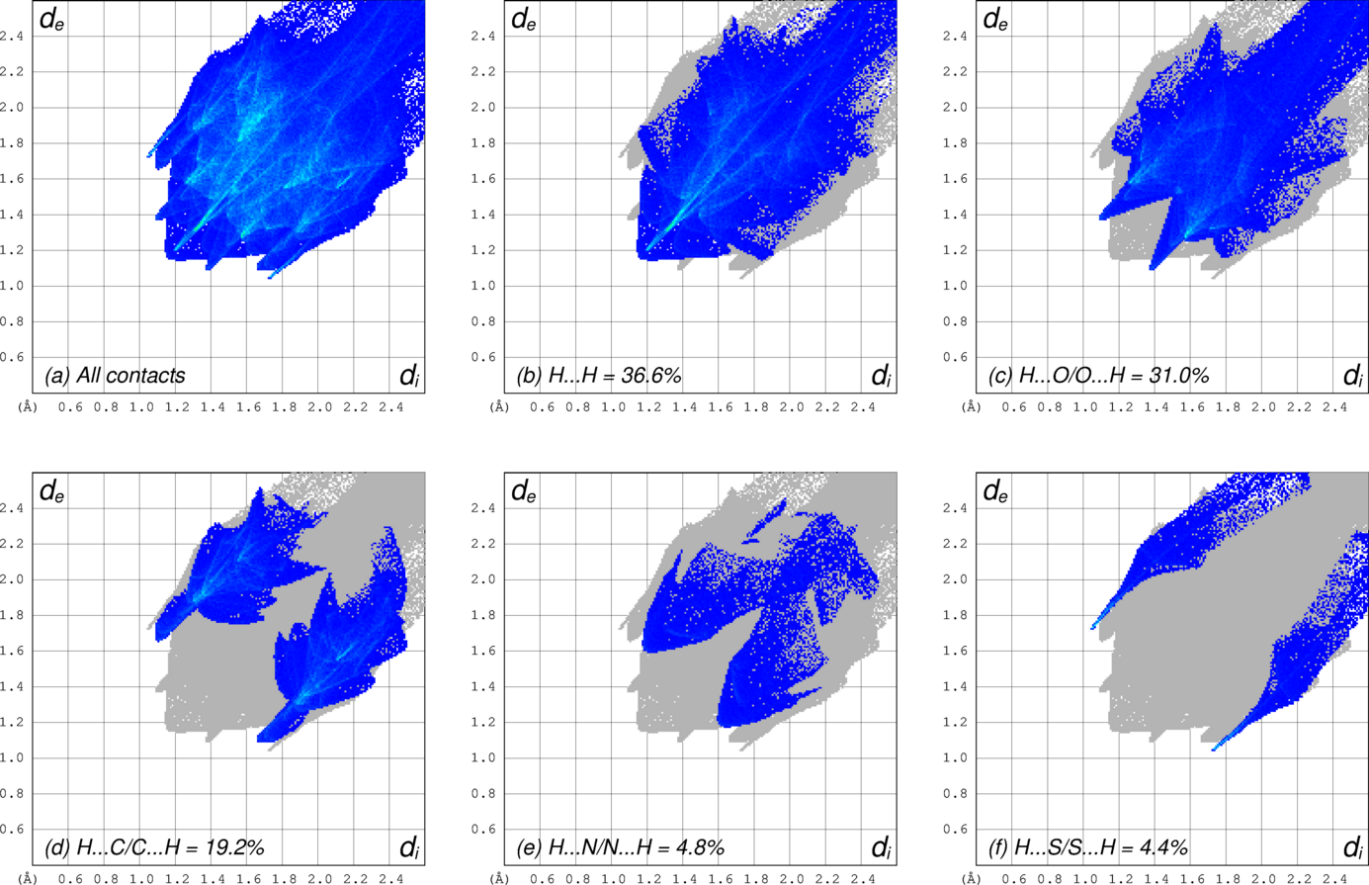
Two-dimensional fingerprint plots from a Hirshfeld surface analysis of **I** showing: (*a*) all contacts; (*b*) H⋯H (36.6%); (*c*) H⋯O/O⋯H (31.0%); (*d*) H⋯C/C⋯H (19.2%); (*e*) H⋯N/N⋯H (4.8%); (*f*) H⋯S/S⋯H (4.4%).

There are no conventional hydrogen bonds in the in the crystal structure of **I**. There are, however, three weak hydrogen-bond-type inter­actions with C—H donors and S or O acceptors (Table 2 ▸). Of these, only the C9—H9*B*⋯O3^iv^ [*d_D⋯A_* = 3.213 (2) Å] and C7—H7*A*⋯S1^iii^ [*d_D⋯A_* = 3.8362 (19) Å] (symmetry codes as per Table 2 ▸) contacts are inter­molecular. The former (and their 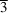-symmetric equivalents) link groups of six mol­ecules into puckered ring assemblies about the *c*-axis, which create and confine the solvent-accessible channels that extend along [001] (Fig. 2 ▸). Attempts to create an unambiguous model for the solvent within these channels were unsatis­factory (see section 6, below). Individual mol­ecules loosely stack into columns that propagate parallel to [001] *via* the C7—H7*A*⋯S1^iii^ (and their symmetry equivalent) inter­actions (Fig. 3 ▸). Adjacent columns are anti­parallel (*i.e.*, along [001] and [00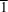]). Two-dimensional fingerprint plots from a Hirshfeld surface analysis conducted using *CrystalExplorer* (Spackman *et al.*, 2021 ▸) show that almost all inter­molecular contacts (∼96% of the total) involve hydrogen. These are shown in Fig. 4 ▸, separated into H⋯H (36.6%), H⋯O (31.0%), H⋯C (19.2%), H⋯N (4.8%), and H⋯S (4.4%), including reciprocal contacts. All other types, *i.e.* those not involving hydrogen, have negligible coverage.

## 4. Database survey

A search of the Cambridge Structural Database (CSD, version 5.45, update of March 2024; Groom *et al.*, 2016 ▸) using a search fragment consisting of just the organic ligand, returned two hits: ZIMNOA (Saeed *et al.*, 2013 ▸), a square-planar Ni^II^ complex that contains two of the ligands and NOJWIV (Kuchar *et al.*, 2019 ▸), a gold complex that has little else in common with **I**. A modified search with the NO_2_ group replaced by ‘any atom’ gave 75 matches. A combined search using this same fragment, but restricted to only trigonal or hexa­gonal crystal systems resulted in four matches: DOVDOK (Barnard & Koch, 2019 ▸), YUFBIK (Bensch & Schuster, 1995 ▸), YIVROM (Mandal & Ray, 2014 ▸), and VEMKIH (Sieler *et al.*, 1990 ▸). These four structures are isotypic to **I**, and share the same space-group symmetry (*P*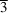). The most similar to **I** are entries DOVDOK and YUFBIK; each have cobalt as the metal centre, with –OMe and –H, respectively, in place of NO_2_. Structures YIVROM and VEMKIH contain iron and ruthenium, respectively, and similar to YUFBIK, have H at the 4-position of the benzene ring. Structures DOVDOK and YIVROM include water in the channels along [001].

## 5. Synthesis and crystallization

Cis-(en)_2_Co(OSO_2_CF_3_)]CF_3_SO_3_ (0.993 g, 1.57 mmol) and *N*,*N*-diethyl-*N*′-[(4-nitro­benzene)(oxo)meth­yl]carbamimido­thio­ate (Weiqun *et al.*, 2003 ▸) (0.524 g, 1.93 mmol) were added to 10 g of sulfolane, stoppered and stirred at room temperature (4 days) resulting in a dark-green solution. Extraction with one 100 mL portion of diethyl ether followed by two 100 mL portions of chloro­form resulted in the formation of a maroon precipitate and dark-green solution. Evaporation of the diethyl ether/chloro­form mixture resulted in deposition of dark-green crystals of the title complex (0.129 g, 9%).

## 6. Data collection, structure solution and refinement

On standard cold-N_2_ gas stream cooling below about 100 K, all crystals of **I** could be indexed as primitive monoclinic, giving cell dimensions of approximately *a* = 16.6, *b* = 9.1, *c* = 44.1 Å, *β* = 100.6°, but many reflections were split and/or streaked, the severity of which varied from crystal to crystal. At room temperature, however, the symmetry was clearly trigonal or hexa­gonal, with sharp diffraction maxima. Attempts to ‘lock in’ the room-temperature structure by rapid cooling in liquid N_2_ and mounting using cryotongs (Parkin & Hope, 1998 ▸) were unsuccessful. One such crystal, however, was monitored on slow warming at about 10° per minute. By 180 K, all splitting/streaking had disappeared. This crystal was used for data collection; details are given in Table 3 ▸.

Structure solution (*SHELXT;* Sheldrick, 2015*a* ▸) and refinement (*SHELXL;* Sheldrick, 2015*b* ▸) were straightforward aside from the presence of severely disordered electron density in the channels running along [001]. Modelling of this diffuse electron density as fractional-occupancy chloro­form was less than satisfactory, perhaps because the presence of other species (*e.g.* water) could not be ruled out [water was modelled in the channels of DOVDOK and YIVROM (see section 4, above)]. For this reason, the *SQUEEZE* routine (van der Sluis & Spek, 1990 ▸; Spek, 2015 ▸) in *PLATON* (Spek, 2020 ▸) was used to factor out the solvent contribution, which amounted to ∼12.5 electrons per asymmetric unit.

All H atoms were found in difference-Fourier maps, but subsequently included in the refinement using riding models, with constrained distances set to 0.95 Å (C*sp*^2^—H), 0.98 Å (*R*—CH_3_) and 0.99 Å (*R*_2_—CH_2_). *U*_iso_(H) parameters were set to values of either 1.2*U*_eq_ or 1.5*U*_eq_ (*R*—CH_3_ only) of the attached atom.

## Supplementary Material

Crystal structure: contains datablock(s) I, global. DOI: 10.1107/S2056989024005449/nx2011sup1.cif

Structure factors: contains datablock(s) I. DOI: 10.1107/S2056989024005449/nx2011Isup2.hkl

CCDC reference: 2361104

Additional supporting information:  crystallographic information; 3D view; checkCIF report

## Figures and Tables

**Table 1:** Selected geometric parameters (Å, °) for **I**

Distances		Angles	
Co1—S1^*sym*^	2.2082 (5)	(S1—Co1—S1)^*sym*^	88.35 (2)
Co1—O1^*sym*^	1.9202 (11)	(O1—Co1—O1)^*sym*^	84.68 (5)
		O1—Co1—S1	95.09 (3)
		O1—Co1—S1^i^	91.87 (4)
		O1—Co1—S1^ii^	176.56 (3)

Symmetry codes: (i) −*y* + 1, *x* − *y* + 1, *z*; (ii) −*x* + *y*, −*x* + 1, *z*; here ‘*sym*’ refers to all crystallographic equivalents about the threefold axis, *i.e.*, (i), (ii), and *x*, *y*, *z*.

**Table 2:** Close contacts (Å, °) for **I**

*D*—H⋯*A*	*D*—H	H⋯*A*	*D*⋯*A*	*D*—H⋯*A*
C12—H12*B*⋯S1	0.98	3.03	3.510 (2)	111.6
C7—H7*A*⋯S1^iii^	0.95	2.91	3.8362 (19)	166.4
C9—H9*B*⋯O3^iv^	0.99	2.53	3.213 (2)	126.3

Symmetry codes: (iii) −*y* + 1, *x* − *y* + 1, *z* + 1; (iv) *x* − *y*, *x*, −*z* + 2.

**Table 3:** Experimental details

Crystal data	
Chemical formula	[Co(C_12_H_14_N_3_O_3_S)_3_]
*M*_r_	899.89
Crystal system, space group	Trigonal, *P* 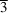
Temperature (K)	180
*a*, *c* (Å)	16.6906 (3), 9.1346 (2)
*V* (Å^3^)	2203.76 (9)
*Z*	2
Radiation type	Cu *K*α
μ (mm^−1^)	4.89
Crystal size (mm)	0.12 × 0.11 × 0.04

Data collection	
Diffractometer	Bruker X8 Proteum diffractometer
Absorption correction	Multi-scan [*SADABS* (Krause *et al.*, 2015 ▸), *XABS2* (Parkin *et al.*, 1995 ▸)]
*T*_min_, *T*_max_	0.580, 0.753
No. of measured, independent and observed [*I* > 2σ(*I*)] reflections	30546, 2682, 2571
*R*_int_	0.038
(sin θ/λ)_max_ (Å^−1^)	0.602

Refinement	
*R*[*F*^2^ > 2σ(*F*^2^)], *wR*(*F*^2^), *S*	0.030, 0.083, 1.08
No. of reflections	2682
No. of parameters	179
H-atom treatment	H-atom parameters constrained
Δρ_max_, Δρ_min_ (e Å^−3^)	0.20, −0.25

Computer programs: *APEX2* (Bruker, 2006 ▸), *SHELXT* (Sheldrick, 2015*a* ▸), *SHELXL2019/3* (Sheldrick, 2015*b* ▸), *XP* in *SHELXTL* (Sheldrick, 2008 ▸), *Mercury* (Macrae *et al.*, 2020 ▸), *SHELX* (Sheldrick, 2008 ▸) and *publCIF* (Westrip, 2010 ▸).

## supplementary crystallographic information

### Tris{N,N-diethyl-N'-[(4-nitrophenyl)(oxo)methyl]carbamimidothioato}cobalt(III) Crystal data

**Table:**

[Co(C_12_H_14_N_3_O_3_S)_3_]	*D*_x_ = 1.356 Mg m^−^^3^
*M**_r_* = 899.89	Cu *K*α radiation, λ = 1.54178 Å
Trigonal, *P*3	Cell parameters from 9548 reflections
*a* = 16.6906 (3) Å	θ = 3.1–68.1°
*c* = 9.1346 (2) Å	µ = 4.89 mm^−^^1^
*V* = 2203.76 (9) Å^3^	*T* = 180 K
*Z* = 2	Shard, dark green
*F*(000) = 936	0.12 × 0.11 × 0.04 mm

### Tris{N,N-diethyl-N'-[(4-nitrophenyl)(oxo)methyl]carbamimidothioato}cobalt(III) Data collection

**Table:**

Bruker X8 Proteum diffractometer	2682 independent reflections
Radiation source: fine-focus rotating anode	2571 reflections with *I* > 2σ(*I*)
Detector resolution: 5.6 pixels mm^-1^	*R*_int_ = 0.038
φ and ω scans	θ_max_ = 68.1°, θ_min_ = 3.1°
Absorption correction: multi-scan [*SADABS* (Krause *et al*., 2015), *XABS2* (Parkin *et al*., 1995)]	*h* = −20→14
*T*_min_ = 0.580, *T*_max_ = 0.753	*k* = −20→20
30546 measured reflections	*l* = −10→10

### Tris{N,N-diethyl-N'-[(4-nitrophenyl)(oxo)methyl]carbamimidothioato}cobalt(III) Refinement

**Table:**

Refinement on *F*^2^	Secondary atom site location: difference Fourier map
Least-squares matrix: full	Hydrogen site location: difference Fourier map
*R*[*F*^2^ > 2σ(*F*^2^)] = 0.030	H-atom parameters constrained
*wR*(*F*^2^) = 0.083	*w* = 1/[σ^2^(*F*_o_^2^) + (0.0455*P*)^2^ + 0.5678*P*] where *P* = (*F*_o_^2^ + 2*F*_c_^2^)/3
*S* = 1.08	(Δ/σ)_max_ = 0.001
2682 reflections	Δρ_max_ = 0.20 e Å^−^^3^
179 parameters	Δρ_min_ = −0.25 e Å^−^^3^
0 restraints	Extinction correction: *SHELXL2019/3* (Sheldrick, 2015b), Fc^*^=kFc[1+0.001xFc^2^λ^3^/sin(2θ)]^-1/4^
Primary atom site location: structure-invariant direct methods	Extinction coefficient: 0.00080 (18)

### Tris{N,N-diethyl-N'-[(4-nitrophenyl)(oxo)methyl]carbamimidothioato}cobalt(III) Special details

**Table:**

Experimental. The crystal was mounted using polyisobutene oil on the tip of a fine glass fibre, which was fastened in a copper mounting pin with electrical solder. It was placed directly into the cold gas stream of a liquid-nitrogen based cryostat (Hope, 1994; Parkin & Hope, 1998). The crystals underwent a reversible phase transition to a triply twinned incommensurately modulated phase when cooled to 90K. Visual inspection of crystal integrity and diffraction quality vs temperature established a safe temperature for data collection of -93° C.
Geometry. All esds (except the esd in the dihedral angle between two l.s. planes) are estimated using the full covariance matrix. The cell esds are taken into account individually in the estimation of esds in distances, angles and torsion angles; correlations between esds in cell parameters are only used when they are defined by crystal symmetry. An approximate (isotropic) treatment of cell esds is used for estimating esds involving l.s. planes.
Refinement. Refinement progress was checked using *PLATO* (Spek, 2020) and by an *R*-tensor (Parkin, 2000). The final model was further checked with the IUCr utility *checkCIF*.

### Tris{N,N-diethyl-N'-[(4-nitrophenyl)(oxo)methyl]carbamimidothioato}cobalt(III) Fractional atomic coordinates and isotropic or equivalent isotropic displacement parameters (Å^2^)

**Table:**

	*x*	*y*	*z*	*U*_iso_*/*U*_eq_	
Co1	0.333333	0.666667	0.48360 (4)	0.03514 (14)	
S1	0.21178 (3)	0.58999 (3)	0.34007 (5)	0.04670 (15)	
O1	0.30064 (7)	0.56544 (7)	0.61574 (12)	0.0381 (3)	
N1	0.14855 (9)	0.45396 (10)	0.55380 (14)	0.0397 (3)	
N2	0.06185 (9)	0.42651 (11)	0.35035 (14)	0.0446 (3)	
N3	0.20338 (13)	0.32053 (16)	1.18453 (19)	0.0637 (5)	
O2	0.16284 (15)	0.23576 (15)	1.18709 (19)	0.0855 (5)	
O3	0.24204 (13)	0.36914 (15)	1.28994 (17)	0.0922 (6)	
C1	0.13955 (11)	0.48454 (12)	0.42182 (16)	0.0385 (4)	
C2	0.22264 (10)	0.49475 (11)	0.63716 (16)	0.0337 (3)	
C3	0.21352 (11)	0.44836 (11)	0.78216 (16)	0.0355 (3)	
C4	0.14649 (12)	0.35663 (13)	0.80514 (18)	0.0442 (4)	
H4A	0.102965	0.323122	0.730224	0.053*	
C5	0.14277 (13)	0.31379 (14)	0.9367 (2)	0.0506 (4)	
H5A	0.098657	0.250373	0.951941	0.061*	
C6	0.20480 (13)	0.36553 (15)	1.04516 (18)	0.0475 (4)	
C7	0.26939 (13)	0.45713 (14)	1.02840 (19)	0.0501 (4)	
H7A	0.309495	0.491487	1.106539	0.060*	
C8	0.27465 (12)	0.49831 (13)	0.89451 (18)	0.0437 (4)	
H8A	0.320351	0.561190	0.879134	0.052*	
C9	−0.00988 (12)	0.34040 (15)	0.4202 (2)	0.0567 (5)	
H9A	−0.071422	0.325873	0.383010	0.068*	
H9B	−0.009034	0.350294	0.527181	0.068*	
C10	0.00396 (16)	0.25957 (16)	0.3914 (3)	0.0696 (6)	
H10A	−0.046291	0.203954	0.437066	0.104*	
H10B	0.063308	0.272139	0.432840	0.104*	
H10C	0.003909	0.249866	0.285617	0.104*	
C11	0.03874 (12)	0.44629 (14)	0.20525 (18)	0.0477 (4)	
H11A	0.002515	0.387563	0.150922	0.057*	
H11B	0.096499	0.485334	0.150052	0.057*	
C12	−0.01665 (15)	0.49554 (18)	0.2149 (2)	0.0637 (6)	
H12A	−0.033471	0.504856	0.116135	0.096*	
H12B	0.020665	0.555658	0.262676	0.096*	
H12C	−0.072966	0.457932	0.272089	0.096*	

### Tris{N,N-diethyl-N'-[(4-nitrophenyl)(oxo)methyl]carbamimidothioato}cobalt(III) Atomic displacement parameters (Å^2^)

**Table:**

	*U*^11^	*U*^22^	*U*^33^	*U*^12^	*U*^13^	*U*^23^
Co1	0.03772 (18)	0.03772 (18)	0.0300 (2)	0.01886 (9)	0.000	0.000
S1	0.0477 (2)	0.0531 (3)	0.0353 (2)	0.0221 (2)	−0.00973 (17)	0.00513 (17)
O1	0.0392 (6)	0.0374 (6)	0.0367 (6)	0.0184 (5)	−0.0083 (4)	−0.0005 (4)
N1	0.0343 (7)	0.0538 (8)	0.0258 (6)	0.0180 (6)	−0.0017 (5)	0.0002 (6)
N2	0.0341 (7)	0.0661 (9)	0.0272 (7)	0.0203 (7)	−0.0042 (5)	−0.0021 (6)
N3	0.0647 (11)	0.0996 (15)	0.0439 (10)	0.0539 (11)	0.0078 (8)	0.023 (1)
O2	0.1145 (15)	0.1025 (14)	0.0634 (10)	0.0722 (12)	0.018 (1)	0.0327 (10)
O3	0.0829 (12)	0.1351 (17)	0.0430 (9)	0.0427 (11)	−0.0153 (8)	0.0233 (10)
C1	0.0334 (8)	0.0541 (9)	0.0284 (8)	0.0220 (7)	−0.0003 (6)	−0.0029 (6)
C2	0.0372 (8)	0.0409 (8)	0.0282 (7)	0.0233 (7)	−0.0021 (6)	−0.0047 (6)
C3	0.0381 (8)	0.0475 (9)	0.0274 (7)	0.0262 (7)	−0.0014 (6)	−0.0024 (6)
C4	0.0418 (9)	0.0528 (10)	0.0331 (8)	0.0201 (8)	−0.0019 (7)	0.0004 (7)
C5	0.0493 (10)	0.0576 (11)	0.0435 (10)	0.0256 (9)	0.0060 (8)	0.0114 (8)
C6	0.0507 (10)	0.0752 (13)	0.0309 (8)	0.0421 (10)	0.0030 (7)	0.0095 (8)
C7	0.0539 (10)	0.0723 (13)	0.0316 (9)	0.0372 (10)	−0.0099 (7)	−0.0056 (8)
C8	0.0472 (9)	0.0519 (10)	0.0354 (9)	0.0273 (8)	−0.0082 (7)	−0.0050 (7)
C9	0.0336 (9)	0.0778 (14)	0.0365 (9)	0.0112 (9)	−0.0016 (7)	−0.0002 (8)
C10	0.0578 (12)	0.0661 (14)	0.0576 (13)	0.0105 (10)	0.0003 (10)	0.0028 (10)
C11	0.0418 (9)	0.0761 (12)	0.0287 (8)	0.0321 (9)	−0.0069 (7)	−0.0065 (8)
C12	0.0608 (12)	0.1016 (17)	0.0469 (11)	0.0541 (13)	−0.0111 (9)	−0.0114 (11)

### Tris{N,N-diethyl-N'-[(4-nitrophenyl)(oxo)methyl]carbamimidothioato}cobalt(III) Geometric parameters (Å, º)

**Table:**

Co1—O1^i^	1.9202 (11)	C4—H4A	0.9500
Co1—O1^ii^	1.9202 (11)	C5—C6	1.380 (3)
Co1—O1	1.9202 (11)	C5—H5A	0.9500
Co1—S1	2.2082 (5)	C6—C7	1.369 (3)
Co1—S1^i^	2.2082 (5)	C7—C8	1.384 (2)
Co1—S1^ii^	2.2082 (5)	C7—H7A	0.9500
S1—C1	1.7284 (17)	C8—H8A	0.9500
O1—C2	1.2606 (19)	C9—C10	1.501 (3)
N1—C2	1.316 (2)	C9—H9A	0.9900
N1—C1	1.347 (2)	C9—H9B	0.9900
N2—C1	1.338 (2)	C10—H10A	0.9800
N2—C11	1.464 (2)	C10—H10B	0.9800
N2—C9	1.478 (2)	C10—H10C	0.9800
N3—O3	1.216 (3)	C11—C12	1.516 (3)
N3—O2	1.226 (3)	C11—H11A	0.9900
N3—C6	1.472 (2)	C11—H11B	0.9900
C2—C3	1.503 (2)	C12—H12A	0.9800
C3—C4	1.388 (2)	C12—H12B	0.9800
C3—C8	1.392 (2)	C12—H12C	0.9800
C4—C5	1.384 (2)		
			
O1^i^—Co1—O1^ii^	84.68 (5)	C5—C4—H4A	119.9
O1^i^—Co1—O1	84.68 (5)	C3—C4—H4A	119.9
O1^ii^—Co1—O1	84.68 (5)	C6—C5—C4	118.31 (18)
O1^i^—Co1—S1	91.87 (4)	C6—C5—H5A	120.8
O1^ii^—Co1—S1	176.56 (3)	C4—C5—H5A	120.8
O1—Co1—S1	95.09 (3)	C7—C6—C5	122.94 (16)
O1^i^—Co1—S1^i^	95.09 (3)	C7—C6—N3	117.99 (18)
O1^ii^—Co1—S1^i^	91.87 (4)	C5—C6—N3	119.06 (19)
O1—Co1—S1^i^	176.56 (4)	C6—C7—C8	118.19 (17)
S1—Co1—S1^i^	88.35 (2)	C6—C7—H7A	120.9
O1^i^—Co1—S1^ii^	176.56 (3)	C8—C7—H7A	120.9
O1^ii^—Co1—S1^ii^	95.09 (3)	C7—C8—C3	120.56 (17)
O1—Co1—S1^ii^	91.87 (4)	C7—C8—H8A	119.7
S1—Co1—S1^ii^	88.35 (2)	C3—C8—H8A	119.7
S1^i^—Co1—S1^ii^	88.35 (2)	N2—C9—C10	112.60 (16)
C1—S1—Co1^ii^	107.73 (5)	N2—C9—H9A	109.1
C1—S1—Co1	107.73 (5)	C10—C9—H9A	109.1
C1—S1—Co1^i^	107.73 (5)	N2—C9—H9B	109.1
C2—O1—Co1^ii^	128.85 (10)	C10—C9—H9B	109.1
C2—O1—Co1^i^	128.85 (10)	H9A—C9—H9B	107.8
C2—O1—Co1	128.85 (10)	C9—C10—H10A	109.5
C2—N1—C1	125.19 (14)	C9—C10—H10B	109.5
C1—N2—C11	123.23 (15)	H10A—C10—H10B	109.5
C1—N2—C9	120.92 (14)	C9—C10—H10C	109.5
C11—N2—C9	115.69 (14)	H10A—C10—H10C	109.5
O3—N3—O2	123.89 (19)	H10B—C10—H10C	109.5
O3—N3—C6	118.5 (2)	N2—C11—C12	111.76 (15)
O2—N3—C6	117.7 (2)	N2—C11—H11A	109.3
N2—C1—N1	114.46 (15)	C12—C11—H11A	109.3
N2—C1—S1	117.16 (12)	N2—C11—H11B	109.3
N1—C1—S1	128.28 (12)	C12—C11—H11B	109.3
O1—C2—N1	131.15 (14)	H11A—C11—H11B	107.9
O1—C2—C3	114.22 (13)	C11—C12—H12A	109.5
N1—C2—C3	114.62 (14)	C11—C12—H12B	109.5
C4—C3—C8	119.62 (15)	H12A—C12—H12B	109.5
C4—C3—C2	121.31 (14)	C11—C12—H12C	109.5
C8—C3—C2	119.07 (15)	H12A—C12—H12C	109.5
C5—C4—C3	120.29 (16)	H12B—C12—H12C	109.5
			
C11—N2—C1—N1	−179.82 (15)	N1—C2—C3—C4	19.9 (2)
C9—N2—C1—N1	4.9 (2)	O1—C2—C3—C8	19.8 (2)
C11—N2—C1—S1	3.5 (2)	N1—C2—C3—C8	−161.13 (15)
C9—N2—C1—S1	−171.79 (14)	C8—C3—C4—C5	−2.7 (3)
C2—N1—C1—N2	173.13 (15)	C2—C3—C4—C5	176.26 (15)
C2—N1—C1—S1	−10.6 (2)	C3—C4—C5—C6	2.5 (3)
Co1^ii^—S1—C1—N2	−178.97 (11)	C4—C5—C6—C7	0.2 (3)
Co1—S1—C1—N2	−178.97 (11)	C4—C5—C6—N3	−178.52 (16)
Co1^i^—S1—C1—N2	−178.97 (11)	O3—N3—C6—C7	17.8 (3)
Co1^ii^—S1—C1—N1	4.85 (17)	O2—N3—C6—C7	−162.28 (18)
Co1—S1—C1—N1	4.85 (17)	O3—N3—C6—C5	−163.37 (19)
Co1^i^—S1—C1—N1	4.85 (17)	O2—N3—C6—C5	16.5 (3)
Co1^ii^—O1—C2—N1	22.6 (2)	C5—C6—C7—C8	−2.5 (3)
Co1^i^—O1—C2—N1	22.6 (2)	N3—C6—C7—C8	176.20 (16)
Co1—O1—C2—N1	22.6 (2)	C6—C7—C8—C3	2.2 (3)
Co1^ii^—O1—C2—C3	−158.52 (10)	C4—C3—C8—C7	0.3 (3)
Co1^i^—O1—C2—C3	−158.52 (10)	C2—C3—C8—C7	−178.67 (15)
Co1—O1—C2—C3	−158.52 (10)	C1—N2—C9—C10	−90.7 (2)
C1—N1—C2—O1	−3.4 (3)	C11—N2—C9—C10	93.7 (2)
C1—N1—C2—C3	177.75 (14)	C1—N2—C11—C12	−90.7 (2)
O1—C2—C3—C4	−159.17 (15)	C9—N2—C11—C12	84.8 (2)

Symmetry codes: (i) −*x*+*y*, −*x*+1, *z*; (ii) −*y*+1, *x*−*y*+1, *z*.

### Tris{N,N-diethyl-N'-[(4-nitrophenyl)(oxo)methyl]carbamimidothioato}cobalt(III) Hydrogen-bond geometry (Å, º)

**Table:**

*D*—H···*A*	*D*—H	H···*A*	*D*···*A*	*D*—H···*A*
C12—H12*B*···S1	0.98	3.03	3.510 (2)	112
C7—H7*A*···S1^iii^	0.95	2.91	3.8362 (19)	166
C9—H9*B*···O3^iv^	0.99	2.53	3.213 (2)	126

Symmetry codes: (iii) −*y*+1, *x*−*y*+1, *z*+1; (iv) *x*−*y*, *x*, −*z*+2.

## References

[bb1] Barnard, I. & Koch, K. R. (2019). *Inorg. Chim. Acta*, **495**, 119019.

[bb2] Bensch, W. & Schuster, M. (1995). *Z. Kristallogr.* **210**, 68–68.

[bb3] Brese, N. E. & O’Keeffe, M. (1991). *Acta Cryst.* B**47**, 192–197.

[bb4] Bruker (2006). *APEX2.* Bruker AXS Inc., Madison, Wisconsin, USA.

[bb5] Dixon, N. E., Jackson, W. G., Lancaster, M. J., Lawrance, G. A. & Sargeson, A. M. (1981). *Inorg. Chem.* **20**, 470–476.

[bb6] Groom, C. R., Bruno, I. J., Lightfoot, M. P. & Ward, S. C. (2016). *Acta Cryst.* B**72**, 171–179. 10.1107/S2052520616003954 PMC4822653 27048719

[bb7] Khan, E., Khan, S., Gul, Z. & Muhammad, M. (2021). *Crit. Rev. Anal. Chem.* **51**, 812–834. 10.1080/10408347.2020.1777523 32571090

[bb8] Krause, L., Herbst-Irmer, R., Sheldrick, G. M. & Stalke, D. (2015). *J. Appl. Cryst.* **48**, 3–10. 10.1107/S1600576714022985 PMC4453166 26089746

[bb9] Kuchar, J., Rust, J., Lehmann, C. W. & Mohr, F. (2019). *New J. Chem.* **43**, 10750–10754.

[bb10] Macrae, C. F., Sovago, I., Cottrell, S. J., Galek, P. T. A., McCabe, P., Pidcock, E., Platings, M., Shields, G. P., Stevens, J. S., Towler, M. & Wood, P. A. (2020). *J. Appl. Cryst.* **53**, 226–235. 10.1107/S1600576719014092 PMC6998782 32047413

[bb11] Mandal, H. & Ray, D. (2014). *Inorg. Chim. Acta*, **414**, 127–133.

[bb12] Parkin, S. & Hope, H. (1998). *J. Appl. Cryst.* **31**, 945–953.

[bb13] Parkin, S., Moezzi, B. & Hope, H. (1995). *J. Appl. Cryst.* **28**, 53–56.

[bb14] Saeed, A., Flörke, U. & Erben, M. F. (2014). *J. Sulfur Chem.* **35**, 318–355.

[bb15] Saeed, S., Rashid, N., Azad Malik, M., O’Brien, P. & Wong, W. T. (2013). *J. Coord. Chem.* **66**, 2788–2801.

[bb16] Sheldrick, G. M. (2008). *Acta Cryst.* A**64**, 112–122. 10.1107/S0108767307043930 18156677

[bb17] Sheldrick, G. M. (2015*a*). *Acta Cryst.* A**71**, 3–8.

[bb18] Sheldrick, G. M. (2015*b*). *Acta Cryst.* C**71**, 3–8.

[bb19] Sieler, J., Richter, R., Hoyer, E., Beyer, L., Lindqvist, O. & Andersen, L. (1990). *Z. Anorg. Allg. Chem.* **580**, 167–174.

[bb20] Sluis, P. van der & Spek, A. L. (1990). *Acta Cryst.* A**46**, 194–201.

[bb21] Spackman, P. R., Turner, M. J., McKinnon, J. J., Wolff, S. K., Grimwood, D. J., Jayatilaka, D. & Spackman, M. A. (2021). *J. Appl. Cryst.* **54**, 1006–1011. 10.1107/S1600576721002910 PMC8202033 34188619

[bb22] Spek, A. L. (2015). *Acta Cryst.* C**71**, 9–18. 10.1107/S2053229614024929 25567569

[bb23] Spek, A. L. (2020). *Acta Cryst.* E**76**, 1–11. 10.1107/S2056989019016244 PMC6944088 31921444

[bb24] Weiqun, Z., Kuisheng, L., Yong, Z. & Lu, L. (2003). *J. Mol. Struct.* **657**, 215–223.

[bb25] Weiqun, Z., Wen, Y., Liqun, X. & Xianchen, C. (2005). *J. Inorg. Biochem.* **99**, 1314–1319. 10.1016/j.jinorgbio.2005.03.004 15917085

[bb26] Westrip, S. P. (2010). *J. Appl. Cryst.* **43**, 920–925.

[bb27] Zahra, U., Saeed, A., Abdul Fattah, T., Flörke, U. & Erben, M. F. (2022). *RSC Adv.* **12**, 12710–12745. 10.1039/d2ra01781d PMC9041296 35496330

